# Understanding and Assessing Cultural Intelligence: Maximum-Performance and Typical-Performance Approaches

**DOI:** 10.3390/jintelligence9030045

**Published:** 2021-09-08

**Authors:** Robert J. Sternberg, Chak Haang Wong, Anastasia P. Kreisel

**Affiliations:** Department of Human Development, College of Human Ecology, Cornell University, Ithaca, NY 14853, USA; cw574@cornell.edu (C.H.W.); apk75@cornell.edu (A.P.K.)

**Keywords:** cultural intelligence, fluid intelligence, intelligence, maximum-performance test, openness to experience, personal intelligence, typical-performance test

## Abstract

Cultural intelligence is one’s ability to adapt when confronted with problems arising in interactions with people or artifacts of diverse cultures. In this study, we conduct an initial construct-validation and assessment of a maximum-performance test of cultural intelligence. We assess the psychometric properties of the test and also correlate the test with other measures with which it might be expected there would be some connection. We found that our test was internally consistent and correlated significantly with maximum-performance tests of abilities but generally less or not at all with typical-performance tests, including cultural intelligence and openness to experience. However, our test appears to be distinct in what it measures from the other tests of cognitive abilities. The results lead us to suggest that cultural intelligence may have both maximum-performance and typical-performance aspects.

## 1. Introduction

Those of us who have traveled abroad all have our funny or even bizarre stories to tell. For the senior author, there was the time in Spain when a musical performance was to be given in his honor, but he rejected it because he did not feel like “Tuna,” not realizing (despite his speaking Spanish) that this was the name of the musical group, not the main course for the dinner. Then there was the time he kept moving away from a conversational partner in Venezuela, who then kept coming closer in a strange kind of ritual dance, because the senior author felt like the conversational partner was making some kind of unwanted advance rather than realizing that the social distance was less than he was used to in the United States. This occurred soon after he took a walk in a neighborhood that seemed friendly and inviting, only for him to learn later that he had walked alone and unprepared in one of the more dangerous neighborhoods in Caracas. Then there was the near-fatal time that he and many other Americans have had visiting England, when he almost got run over by a bus because he looked the wrong way when crossing the street, having forgotten that, in the UK, cars and buses are to be found on the left, not the right side of the road.

Successfully visiting a foreign country requires a kind of intelligence that does not seem quite to be captured by traditional notions of intelligence (e.g., Carroll 1993; McGrew 2005). Yes, one has to adapt to the environment (Binet and Simon 1916; Intelligence and Its Measurement: A Symposium 1921; Sternberg 2021; Wechsler 1940), but the adaptation is to an environment that may be quite different in kind from that to which one is accustomed. In more extreme cases, one may end up in prison or dead for saying or doing the “wrong” thing, without having had even a clue in advance that what one said or did was illegal, and in some cases, perilously dangerous to the actor.

The idea that there may be different kinds of intelligence is scarcely new. Gardner (1983, 2011) based a whole theory on the idea of multiple intelligences, and a recent handbook on intelligence (Sternberg 2020b) contains chapters on Gardner’s multiple intelligences (Kornhaber 2020), as well as on collective intelligence (Malone and Woolley 2020), consumer and marketer intelligence (Sujan and Sujan 2020), emotional intelligence (Rivers et al. 2020), leadership intelligence (Boyatzis 2020), mating intelligence (Geher et al. 2020), practical intelligence (Hedlund 2020), social intelligence (Kihlstrom and Cantor 2020), successful intelligence (Sternberg 2020a), and most relevantly for present purposes, cultural intelligence (Ang et al. 2020).

Cultural intelligence is one’s ability to adapt when confronted with problems arising in interactions with people of diverse cultures. The concept of cultural intelligence (CI) (or what Ang and her colleagues refer to as “CQ”) is based on the idea that acting intelligently in diverse cultures may require more than general intelligence and its subfactors (Ang et al. 2020). Earley and Ang (2003) appear to have introduced the concept. The construct addresses the question of why some people more easily and effectively adapt their views and behaviors across cultures than do others (Ang et al. 2020; Van Dyne et al. 2010). Earley and Mosakowski (2004) suggested that “a person with high cultural intelligence can somehow tease out of a person’s or group’s behavior those features that would be true of all people and all groups, those peculiar to this person or this group, and those that are neither universal nor idiosyncratic” (p. 139). The concept has become prominent in the psychological literature, primarily by Soon Ang and her colleagues in various articles and book chapters (e.g., Ang et al. 2006, 2007, 2015).

Ang and her colleagues (e.g., Ang et al. 2020) have suggested a four-factor model of cultural intelligence: metacognitive CI, cognitive CI, motivational CI, and behavioral CI. According to Ang and colleagues (2020), these factors can be further subdivided into what the researchers refer to as “subdimensions”: planning, awareness, and checking, for metacognitive CI; culture-general knowledge and context-specific knowledge, for cognitive CI; intrinsic interest, extrinsic interest, and self-efficacy to adjust, for motivational CI; and verbal behavior, nonverbal behavior, and speech acts, for behavioral CI.

In at least some of their work, Ang and her colleagues (e.g., Ang et al. 2020) have taken a “typical-performance” approach to the measurement of CI. That is, the measure they use is like a personality inventory, with participants evaluating themselves on questions concerning, for example, their planning for cross-cultural meetings or how they react in cross-cultural situations (Cultural Intelligence Center 2011). Typical-performance items measure test-takers’ implicit theories of themselves—their self-perceptions of what they are like or what they do or would do. The items do not necessarily measure what people actually do or would do in the situations about which the questions are asked. Indeed, most of us may know what we think we would do in a given situation, but do not know what we actually would do.

Drawing upon the theory of successful intelligence (Sternberg 2020a), we have taken a different but complementary approach, namely, one of measuring maximum performance. Historically, typical- and maximum-performance tests of constructs, such as of emotional intelligence (Rivers et al. 2020) or of wisdom (Sternberg and Glück forthcoming), have yielded somewhat different results and scores that correlate minimally, if at all, with each other. This is not to say that one is valid and the other, invalid. Rather, it appears that the maximum- and typical-performance measures assess different aspects of a phenomenon.

The theory of successful intelligence would view cultural intelligence as drawing particularly upon practical, creative, and analytical aspects of intelligence. Practical intelligence itself is based largely upon tacit knowledge, or the knowledge one needs to succeed in everyday situations that one is not explicitly taught and that often is not even verbalized (Sternberg 2020a). Practical intelligence is typically measured by situational-judgment tests that put a respondent in the position of a person confronting a practical problem that they need to solve. Their response is the free-response solution to the practical problem (Sternberg et al. 2000). In their past research, Sternberg and colleagues found measures of practical intelligence, as assessed by tacit knowledge in a professional domain, to correlate with each other, but not so much to correlate with conventional tests of abstract-analytical abilities (i.e., fluid intelligence—Sternberg et al. 2000).

Cultural intelligence, however, also draws upon abstract analytical abilities because one has to analyze situations that, unlike many practical problems, are rather removed from one’s everyday experience. Cultural intelligence draws on creative abilities because the problems one confronts are more novel than one would confront in typical tests as well as life situations. We did not measure creative abilities in the present study, however.

In this study, we sought to begin construct-validation of a cultural-intelligence test. This work is only a beginning, not a complete construct-validation of the test. The test is based, from intelligence theory, on Sternberg’s (2020a) augmented theory of successful intelligence, according to which intelligence comprises creative, analytical, practical, and wisdom-based aspects. The situations presented in our test are novel and open-ended, thus requiring creative thinking to produce what, for the participants, are novel and useful responses, given the unusual situations presented. The test problems require participants to think analytically, that is, analyzing the situations presented in the scenarios in order to understand what is going on and what particular actions mean. The problems require a practical response in that the response has to be adaptive to the everyday constraints presented in the problem. The problems require creativity, because they are quite novel. And the problems require wisdom, in that the goal of the hypothetical individual is, in the business section, to get a memorandum of agreement signed that will benefit all parties, and in the leisure section, to enjoy oneself without imposing on others.

In terms of Ang et al.’s (2020) theory of cultural intelligence, our test measures primarily metacognitive and cognitive aspects of cultural intelligence, the two aspects most relevant to maximum-performance measurement. The problems present cognitive challenges and require metacognitive or metacomponential processes, such as recognizing the existence of a problem and then defining exactly what the problem is. Our tests do not directly measure motivation to engage with others from other cultures and do not measure the behavior individuals would actually exhibit if they were in the situations that are described.

Here is an example of how the scenarios sample the elements of the augmented theory of successful intelligence (Sternberg 2020a) with a randomly selected item:

*“7. You are negotiating with a high-level official in the organization with which you need to sign an agreement. No matter what you say, she seems to disagree. You feel that the negotiation is not going well and that it really doesn’t matter what you say, she will disagree. You really want and need for this negotiation to succeed. What would you do?”*

As defined by the theory, creativity is involved in dealing with a relatively novel situation and in generating responses that are divergent and potentially useful under the circumstances. Analytical skills are involved in figuring out why the high-level official seems to keep disagreeing. Practical skills are involved in figuring out solutions that will attain the desired outcome. Wisdom is involved in trying to achieve a common good for your business and for the business with which you are negotiating, as otherwise it will be hard to reach any agreement at all. The same sampling of skills is involved in solving almost all the items. However, we have not attempted separate scores because, according to the theory, the processes are highly intertwined rather than separate.

Our measure is maximum-performance in that it asks what the test-taker would do in the various situations to achieve the best outcome. Of course, responses are not necessarily what the participant really would do; rather, they are hypothetical best responses. They represent the kind of tacit knowledge found in tests of practical intelligence (Hedlund 2020). Further, any best response is likely to be a compromise between different constraints. That is, there may be no single best response that optimizes all possible aspects of a situation. Cultural intelligence has both maximum-performance and typical-performance aspects, and if the literature on emotional intelligence is any guide (Rivers et al. 2020), one would not expect much, or perhaps any, correlation between the two domains.

Sackett et al. (1988) have suggested several criteria for designating a test as “maximum performance.” These criteria are that there is (a) explicit awareness that one is being evaluated; (b) awareness and acceptance of the requirement to maximize effort; (c) a time constraint on performance (it is over a short enough period of time) so that the participants can maintain their attention and stay focused on the goal of the task. With regard to these criteria, (a) all the participants signed an informed-consent form showing their knowledge that they would be evaluated; (b) all were aware of the requirement to maximize effort, although there is no guarantee that they did so; and (c) the time limit was well shorter than that on many standardized maximum-performance tests, such as the SAT and the ACT. Thus, we believe these criteria were met.

One could argue that, on a maximum-performance test, one should ask something like “What should you do?”, whereas on a typical-performance test one should instead ask “What would you do?” (McDaniel et al. 2007). We have a very different take on this issue. According to dictionary.com, “should” is typically used to express “duty, propriety, or expediency.” For example, “You should go to church” is a way of saying that going to church is the right thing for you to do. In our view of cultural intelligence, the word “should” would have compromised the construct-validity of the test, as it would have created a situation where participants were trying to figure out the tester’s moral or ethical system of what the tester believes the participant should have done in the given situation. We are not interested in a test that reflected test-wiseness (“what was the examiner thinking?”) or the test-taker’s sense of what would be the moral thing to do. Rather, we were interested in what a test-taker facing a challenging situation actually thought they would do in a challenging situation.

We sampled behavior requiring cultural intelligence (Wernimont and Campbell 1968). The test certainly does not provide a complete sampling. Moreover, the situations are designed to be challenging and puzzling ones. Easily managed situations with easy-to-understand people would not provide much in the way of individual differences for testing cultural intelligence.

The test is not purported to be a full measure of cultural intelligence. Rather, it samples the kinds of situations one might encounter in business or leisure settings

In this study, we correlated our test against measures of abstract analytical reasoning (fluid intelligence), practical intelligence (as measured by a “personal intelligence” test), openness to experience, and cultural intelligence as measured by a typical-performance measure.

There were two subscales in our cultural-intelligence scale—one concerning business-related situations and one concerning leisure-related situations. Our hypothesis was that our two cultural-intelligence subscales would be strongly correlated, as these two subscales are both hypothesized to measure cultural intelligence via maximum performance. Because cultural intelligence would seem to draw on both analytical (fluid) intelligence and practical (personal) intelligence, we also expected significant correlations with the measures of these two constructs.

We also expected weak but significant correlations with the typical-performance measure of cultural intelligence and with the typical-performance-based measure of openness to experience. However, our confidence in these hypotheses was lower because, in previous literature concerning wisdom (see Kunzmann 2019; Webster 2019) and emotional intelligence (see Rivers et al. 2020), maximum- and typical-performance measures have shown, at most, minimal correlations. Thus, it may be simply that maximum- and typical-performance measures assess different aspects of psychological phenomena.

## 2. Method

### 2.1. Participants

A total of 109 undergraduate and graduate students enrolled at Cornell University participated in the online data collection. In all, 29 of them were male, 77 of them were female, 1 of them indicated other, and 2 of them did not indicate their gender. The average age of the participants was 19.90 years, with a standard deviation of 1.51. The racial composition was 33.0% White or Caucasian, 41.3% Asian and Asian American, 6.4% Black or African American, 11.9% Hispanic or Latino, 4.6% two or more races, and 2.8% preferring not to answer.

### 2.2. Materials

There was a total of eight assessments in the form of an online survey on Qualtrics: two psychometric assessments, which included Letter Sets and Figure Classification; two maximum-performance Cultural Intelligence Tests we created, in which one was related to a business trip and the other one was related to a leisure trip; a typical-performance Cultural Intelligence Scale (CQS); an Openness to Experience scale; a Test of Personal Intelligence Mini—12; and a demographic questionnaire.

**Psychometric Assessments.** The two psychometric assessments included in this study were: (1) Letter Sets, in which participants crossed out one set of letters that did not fit in with the other four sets of letters; and (2) Figure Classification, in which participants looked for a figural property that was the same among the three figures in any one group, as well as for a property that made the groups differ from one another.

The psychometric assessments were adapted from Kit of Factor-Referenced Cognitive Tests (Ekstrom et al. 1976) and measured largely fluid intelligence. While the Letter Sets had 15 items and a seven-minute time limit, the Figure Classification had 14 stems with 8 items per stem and an eight-minute time limit. The items were scored based on the number of correct answers, and one point was scored for each correct answer.

**Maximum-Performance Cultural Intelligence Test (CI).** We created two versions of the Cultural Intelligence Test. One was related to a business trip **(CIB)** and one was related to a leisure trip **(CIL)**. In both versions of the Cultural Intelligence Test, we constructed a number of simulated real-word scenarios that one might encounter in different cultural contexts, and we asked the participants what they would do to deal with certain challenges when traveling to a new cultural environment. For example, an item from the CIB was:

*“You have important sensitive and confidential information to convey back to your superiors. When you go down to the receptionist, you are told that there is no problem. You can use a hotel computer, which is connected to the Worldwide Web. Or, she says, you can use the phone in your room and she can connect you to wherever you need to be connected. She then leads you to a computer in the business center. What would you do?”*

Participants previously had been informed that they could not, themselves, establish a personal Internet connection (see the Appendix A.1 for the full item and its context).

There were 10 items for the CIB and 9 items for the CIL. Each item was graded twice by two different graders, and the final score was decided by both graders. The scoring was based on a 5-point scale, in which 1 indicated a poorly answered item and 5 indicated a thoroughly answered item. The grading rubric for the Cultural Intelligence Tests (both CIB and CIL) was as follows:

0 Point: No answer/blank
1 Point: Provided one plausible response with no/vague further explanation, for example, “I would go to a hospital.”
2 Points: Provided one or two plausible responses with some explanations, for example, “I would go use hand gestures to indicate my illness and ask for a map to find a hospital.”
3 Points: Provided two or more plausible responses with more elaborated explanations, for example, “I would first do…then…if something went wrong, I would…”
4 Points: Provided three or more plausible responses with elaborated explanations.
5 Points: Provided three or more plausible (and novel/unique) responses with specific and detailed explanations.

No 5s were given in this sample. The highest given score was 4, and the lowest score was 1.

For the complete set of items, please see Appendix A.1 (business version) and Appendix A.2 (leisure version). Two raters, who were blind to each other’s ratings, yielded an inter-rater reliability (intraclass correlation) of 0.99+, but this high reliability may reflect the fact that the graders jointly developed the rubric. It thus would remain to be seen whether raters who did not develop the rubric could use it as reliably.

**Typical-Performance Cultural intelligence Scale (CQS).**Van Dyne et al. (2008) completed several studies to develop, validate, and cross-validate the Cultural Intelligence Scale (CQS). The scale is a self-reported (typical-performance), previously validated test of cultural-related knowledge and capabilities, such as knowing a different culture’s values, dealing with cultural adjustment, and adjusting cultural knowledge. An example of an item, presented here with permission of the authors, is: “I check the accuracy of my cultural knowledge as I interact with people from different cultures.” Such items measure the test-taker’s communicated implicit theories of what they would do in particular situations. The CQS measures metacognitive, cognitive, motivational, and behavioral aspects of cultural intelligence.

The scale uses a 7-point Likert scale (from 1 = Strongly Disagree to 7 = Strongly Agree), and higher total scores indicate higher measured cultural intelligence. However, the scale does not involve any actual cultural interactions or scenarios that people encounter in real life. Rather, the participants characterize what they are like in their cross-cultural interactions.

**Openness to Experience (OE).** The typical-performance Openness to Experience scale was adapted from the Big Five Inventory Personality scale (Johnson 2014). There was a total of 24 items, and it used a 5-point Likert scale (1 = Very Inaccurate and 5 = Very Accurate). The scale asked participants to indicate their level of agreement with a series of statements that describe who they are and what their attitudes are toward events in daily life.

**Test of Personal Intelligence Mini—12 (TOPI).** The Test of Personal Intelligence was developed, validated, and tested by Mayer et al. (2018). The TOPI contains 12 items, and it was a maximum-performance problem-solving ability-based test with correct or incorrect answers. After reading a short statement, the participants were asked to choose one out of four answers that they believed was correct.

**Demographic Questionnaire.** The demographic questionnaire assessed variables including age, gender, ethnicity, year at Cornell, SAT and ACT scores (as available), Cornell GPA, first language, number of cross-cultural experiences in years, number of different countries the participants had visited, and the amount of contact the participants have had with people of other cultures.

## 3. Design

The design of this study was correlational. The dependent variables of most interest were the standardized test scores (Letter Sets, Figural Classification, SAT score, ACT score, Cultural Intelligence Scale (CQS) score, Openness to Experience (OE) score, and the Test of Personal Intelligence Mini—12 (TOPI) score) and the independent variables were the Cultural Intelligence Tests (CI) scores for the Business (CIB) and Leisure (CIL) subscales.

### Procedure

The whole study survey was presented online on a Qualtrics platform. The participants were recruited through an on-campus online subject pool system, SONA. Participants were first asked to read and sign the informed consent form; then, they proceeded to the two psychometric assessments. The two assessments had a seven-minute and eight-minute time limit, respectively. The online survey automatically moved to the next page when the time was up. After completion of Figure Classification, the participants had no time limit to finish the rest of the survey, with the materials in the following order: Cultural Intelligence Tests—Business (CIB), Cultural Intelligence Tests—Leisure (CIL), Cultural Intelligence Scale (CQS), Openness to Experience (OE), the Test of Personal Intelligence Mini—12 (TOPI), and a demographic questionnaire. The participants were asked to read the debriefing form after completing the whole survey. The entire sessions lasted no longer than 1.5 h.

## 4. Results

Our report of results is divided into three major sections: basic statistics, correlations, and factor analyses.

### 4.1. Basic Statistics

Table 1 shows the basic statistics for our study of cultural intelligence. As can be seen by the mean ACT and SAT scores, ours was an above-average population of college students in terms of the kinds of academic skills measured by these tests. However, there was quite a bit of variation around the mean, so that we actually had a fair amount of range, for example, with standard deviations of 85 for SAT Reading and 88 for SAT Math (compared with population standard deviations of about 100. (Standard deviations vary by states, and in some states are in the 80s: https://nces.ed.gov/programs/digest/d17/tables/dt17_226.40.asp).

Table 2 shows the results of analyses of variance for sex. (The *N’*s were not large enough for ethnicity analyses to be meaningful). On the ACT, women outperformed men (*p* < 0.05).

Table 3 shows the internal-consistency reliabilities of the various tests (as measured by coefficient alpha). The two parts of our Cultural Intelligence Test had reliabilities of 0.79 (business) and 0.77 (leisure), with a combined reliability of 0.87. These reliabilities were in the same ballpark as some of the other measures we used, but the reliabilites for the two psychometric tests were higher. However, the coefficient alphas for the psychometric tests (.83 for Letter Sets, 0.95 for Figure Classification) may have been partially inflated because the tests were speeded.

### 4.2. Intercorrelations

Table 4 shows intercorrelations among the various measures in the study.

The intercorrelation between the Business and Leisure subtests of our Cultural Intelligence Test was 0.74 (*p* < 0.01). These tests clearly are highly interrelated, as would be expected, as they were both designed to measure cultural intelligence, albeit in different domains of life pursuits.

The correlations with the standardized admissions tests were generally weak. Considering self-reported SAT-Reading, SAT-Math, and ACT, the median correlation was 0.21. Only the correlation of the Cultural Intelligence Leisure measure was statistically significant, at 0.28 (*p* < 0.05). The correlations with the fluid-intelligence measures—Letter Series and Figure Classifications—were higher with, however, a median of 0.42 for Letter Series and 0.34 for Figure Classifications. All the correlations were statistically significant (*p* < 0.01). Our tests therefore show significant relationships with fluid-ability measures, although at levels far below the reliabilities of the tests. Our Cultural Intelligence Test also correlated significantly with the maximum-performance Test of Personal Intelligence (TOPI), 0.31 (*p* < 0.01) for each section.

Our Cultural Intelligence Test generally did not correlate significantly with the typical-performance measure of Cultural Intelligence (CQS), with a median correlation for our two subscales of 0.12. (There was one significant correlation, *p* < 0.05, with the Motivational Subscale.) We obtained a pattern of meaningful correlations neither with overall score nor with dimensional scores. As is true with measures of emotional intelligence (Rivers et al. 2020) and wisdom (Sternberg and Glück forthcoming), typical- and maximum-performance measures appear to measure quite different things. The typical-performance Cultural Intelligence Test (CQS) also did not correlate significantly with any of the fluid-intelligence measures or with the Test of Personal Intelligence (TOPI). However, it did show a significant correlation of 0.26 (*p* < 0.01) with the Openness to Experience (OE) measure, which our measure did not show. Thus, the typical-performance measures generally correlated with each other, and the maximum-performance measures also correlated with each other.

Our measure of Cultural Intelligence did not correlate significantly with reported cultural experiences. The typical-performance CQS, however, did correlate significantly with 0.31 (*p* < 0.01) with years of cross-cultural experience.

### 4.3. Principal-Component and Factor Analyses

Table 5, Table 6, Table 7 and Table 8 show rotated principal—components and principal-factor analyses for different combinations of tests.

Table 5 shows principal-components analysis for Letter Sets, Figure Classification, the two subscales of our maximum-performance Cultural-Intelligence Test (CIB and CIL), the typical-performance Cultural Intelligence Scale (CQS), the Openness to Experience Scale (OE), and the Test of Personal Intelligence (TOPI). These results show that the maximum-performance tests (Letter Sets, Figure Classification, CIB, CIL, TOPI) loaded on one scale, and the typical-performance tests (CQS, OE) on the other.

Table 6 shows the principal-factor analysis for the same tests, with essentially the same results. Thus, the method of factor analysis did not matter.

Table 7 shows principal-components analysis for our cultural-intelligence subscales (CIB and CIL), the typical-performance CQS and OE, and the maximum-performance TOPI and SAT/ACT (combined). The first rotated principal component comprises our two cultural-intelligence subscales. The second rotated principal component comprises the CQS and the OE scales, the two typical-performance measures, and the last factor comprises the TOPI and the SAT/ACT, both maximum-performance measures. The Test of Personal Intelligence, therefore, appears to be closer to the standard aptitude test used for college admission than do our measures, offering some discriminant validity for our subscales.

Table 8 shows a principal-factor analysis of the same scales with the same results, again showing that method of factor analysis essentially made no difference.

We also factor-analyzed the individual items of our cultural-intelligence test. Although we obtained five factors with Eigenvalues greater than 1, only one factor had an Eigenvalue greater than 1.5, and only that first factor was interpretable as a general factor for the test. That factor had an Eigenvalue of 5.9 and accounted for 31% of the variance in the data. Because the test was highly internally consistent, with a coefficient alpha of 0.87, we did not pursue the separate factors from the item-based principal-components and principal-factor analyses.

## 5. Discussion

Our study presents a first step toward construct validation of a new maximum-performance test of cultural intelligence. The results suggest that the test has relatively high internal-consistency reliability, can be scored with exceptionally high inter-rater reliability, and is valid when correlated against aspects of abilities that one might expect to be related to cultural intelligence, such as analytical and practical ones. The test generally does not show significant correlations with a typical-performance test of cultural intelligence or with a measure of openness to experience, although the latter two correlated significantly with each other. We also discovered that maximum-performance assessments of cultural intelligence in the business and leisure domains are highly correlated with each other. Whether the two aspects of cultural intelligence factor with each other alone or with cognitive-ability tests depends on which cognitive-ability tests are used: They factored alone when the self-reported SAT/ACT was used, but with cognitive-abilities tests when Letter Sets and Figure Classification were used. (A factor analysis with all the tests together was unsatisfactory because of insufficient *N* when listwise deletion of cases was used.). A factor analysis of items failed to reveal more than one psychologically meaningful factor for maximum-performance measurement of cultural intelligence.

The results of our research suggest, at least initially, that cultural intelligence, much like emotional intelligence (Rivers et al. 2020) and wisdom (Kunzmann 2019; Webster 2019), and likely other constructs as well, such as creativity (Plucker et al. 2019; Sternberg 2017), can be conceptualized as having both maximum-performance and typical-performance aspects. Maximum-performance tests measure an ability-like aspect, as is measured by conventional tests of abilities. Typical-performance tests measure an attitudinal aspect, as is measured by conventional personality tests. Thus, the ability component of cultural intelligence correlates with tests of fluid and practical cognitive abilities, whereas the personality component correlates with a test of openness to experience. The two kinds of tests should not be viewed as being in competition with each other, as when one asks which is the “true” measure of a construct, but rather as measuring complementary aspects of the construct, in this case, cultural intelligence. To interact intelligently with people of other cultures, one needs to have an attitude of wanting to engage in an open and reflective way with the people of the other cultures, and one also needs the ability to do so.

Our initial construct-validation was, of course, incomplete. It did not include a measure of creative intelligence, which might also be expected to show some significant correlation with cultural intelligence. More importantly, it was conducted only on a sample of undergraduate students at a selective university in the northeast of the United States. We are currently making plans to do a further construct validation involving participants from Asian and European countries, but such a further study will take a long time to conceive and execute due to needs to ensure that the test is translated and also, as necessary, made appropriate in both construction and scoring for participants in a variety of cultures.

One might be concerned that our scoring seems to have emphasized quantity rather than quality of responses. This is not the case. When one is unsure of the cultural customs, language, and general expectations of a culture, there is no one knowable “correct” response. Often, one has to try multiple responses until one figures out exactly how to handle a situation. The rigid individual who can provide only one response and then hopes for the best is likely to fare poorly in inter-cultural situations. Ideally, one perhaps tries a pre-potent response, but then also has backup responses in case the first, second, or even third response does not work. For example, if trying to get directions, it often takes several tries to get the person from another culture with whom one is communicating to understand what is being asked. This is not a matter merely of scoring for quantity. It is a matter of scoring for what cultural intelligence is—a flexibility of responses that recognizes the need for a variety of perhaps diverging responses until one achieves the desired effect. In real-life specific situations, it is intrinsic to cultural intelligence that one have a series of backup responses until the desired response, or something close to it, is elicited. If a test-taker needs to be told this, the test-taker lacks cultural intelligence. We would not want to tell respondents to give a variety of responses, because this would essentially reduce the ecological validity of the test.

Our study had various limitations that we are addressing in current research and will continue to address in future research. It therefore may be useful to enumerate the aspects that we are addressing in this further research. First, the absence of sample items created a situation wherein some participants may not have known exactly what we expected. This could have been a source of individual differences. Second, participants did not know the grading criteria, although it is also possible that figuring out what is expected is part of cultural intelligence. This lack of knowledge of grading criteria is true for most tests—for example, the Torrance Tests of Creative Thinking, the SAT, the ACT, IQ tests, and most statewide achievement tests, and other tests also do not specify criteria for grading. However, participants certainly could benefit by knowing what these criteria are. Third, our participants were all from a selective university in the northeast of the United States. A broader range of participants obviously is needed. Fourth, some of our test items might have suggested a negative view on our part of people in far-flung countries. The kinds of situations we described are ones that can occur in any country, but there seems to be a need for more test items that convey a more positive impression of individuals from diverse cultures. Fifth, the test measures a wide variety of skills, and the skills have not yet been psychometrically or experimentally separated. For example, some of the skills that go into responses are perspicacity regarding inter-cultural situations, verbal fluency, coping with adverse situations, and prior familiarity with challenges of intercultural contact. Most ability tests measure a variety of skills (e.g., mental speed, prior learning, reasoning skills, motivation, test-wiseness), and ultimately, one would want to use some kind of further analysis to separate them out on our test. Sixth, there is a need for a broader range of raters of responses. Finally, we acknowledge that there are no predictive-validity data to address whether our measures predict actual future behavior—we did concurrent validation only. We are addressing *all* these issues in forthcoming research, although the range of issues is such—for example, cross-cultural testing—that it will probably be a matter of years before all the issues are fully addressed. We present this study only as our entrée into testing of cultural intelligence through maximum-performance measurement and acknowledge that there are many issues yet to be resolved. We also invite others who wish to explore our measures to do so, if they wish.

Today, it is difficult to get through one’s life without interacting with people from a variety of cultures. A test of cultural intelligence thus seems like a useful kind of test to have in this day and age. But the test is useful not only for assessment but also potentially for instruction. The problems can serve as situational judgments that can be presented to young people who are learning to engage with people of diverse cultures, so that they can think through the possible responses to challenges they will later face in their interactions across cultural contexts. Thus, our hope is that such a test ultimately will be as useful for training as for assessment purposes.

Whatever may be the uses of the test, we believe the results reinforce the usefulness of a broad rather than a narrow conception of intelligence. We do not question the psychological status or usefulness of general intelligence, but we do believe that intelligence is more variegated than traditional models might suggest. One of these forms of variegation, we suggest, is cultural intelligence.

## Figures and Tables

**Table 1:** Mean scores and standard deviations.

	Mean	Standard Deviation	*N*
Age	19.90	1.51	108
ACT Score	31.66	3.37	61
SAT Reading Score	700.63	85.21	64
SAT Math Score	730.77	87.61	65
SAT to ACT conversion	31.26	4.06	99
Cumulative College GPA	3.59	0.35	82
Letter Sets total score	10.69	3.36	109
Figure Classification total score	62.34	19.71	109
Cultural Intelligence—Business	19.16	3.79	109
Cultural Intelligence—Leisure	17.97	3.33	109
Total Cultural Intelligence Score	37.13	6.65	109
CQS	86.44	17.28	109
CQS Dimension 1 MC	19.89	4.13	109
CQS Dimension 2 COG	20.95	7.18	109
CQS Dimension 3 MOT	22.80	5.55	109
CQS Dimension 4 BEH	22.80	6.02	109
Openness to Experience	74.50	10.74	109
TOPI	9.61	2.57	109
CCE	7.00	7.54	99
Different Country Visited	6.57	4.59	106

Note: CQS = Van Dyne et al. Cultural Intelligence Scale; Four dimensions of CQS are (1) Metacognitive, (2) Cognitive, (3) Motivational, and (4) Behavioral; TOPI = Test of Personal Intelligence; CCE = Cross Cultural Experience in Years.

**Table 2:** Gender-based Analysis of Variance.

			Sum of Squares	df	Mean Square	F	Sig.
ACT * Gender	Between Groups	(Combined)	74.92	2	37.46	3.55	0.04
	Within Groups		602.06	57	10.56		
	Total		676.98	59			
SATReading * Gender	Between Groups	(Combined)	17,732.94	2	8866.47	1.22	0.30
	Within Groups		437,165.48	60	7286.09		
	Total		454,898.41	62			
SATMath * Gender	Between Groups	(Combined)	21,841.75	2	10,920.88	1.42	0.25
	Within Groups		469,044.19	61	7689.25		
	Total		490,885.94	63			
SAT to ACT conversion * Gender	Between Groups	(Combined)	39.69	2	19.84	1.20	0.31
	Within Groups		1575.41	95	16.58		
	Total		1615.10	97			
GPA * Gender	Between Groups	(Combined)	0.19	2	0.09	0.75	0.47
	Within Groups		9.81	78	0.13		
	Total		10.00	80			
Letter Sets total score * Gender	Between Groups	(Combined)	5.48	2	2.74	0.27	0.76
	Within Groups		1054.12	104	10.14		
	Total		1059.61	106			
Figure Classification total score * Gender	Between Groups	(Combined)	312.59	2	156.29	0.40	0.67
	Within Groups		40,771.00	104	392.03		
	Total		41,083.59	106			
CIB total score * Gender	Between Groups	(Combined)	17.37	2	8.68	0.62	0.54
	Within Groups		1449.77	104	13.94		
	Total		1467.14	106			
CIL total score * Gender	Between Groups	(Combined)	0.13	2	0.07	0.01	0.99
	Within Groups		1115.53	104	10.73		
	Total		1115.66	106			
CI total score * Gender	Between Groups	(Combined)	20.48	2	10.24	0.24	.79
	Within Groups		4439.07	104	42.68		
	Total		4459.55	106			
CQS total score * Gender	Between Groups	(Combined)	937.06	2	468.53	1.61	0.20
	Within Groups		30,193.16	104	290.32		
	Total		31,130.22	106			
CQS Dimension 1 MC * Gender	Between Groups	(Combined)	66.42	2	33.21	1.99	0.14
	Within Groups		1731.82	104	16.65		
	Total		1798.24	106			
CQS Dimension 2 COG * Gender	Between Groups	(Combined)	249.45	2	124.72	2.47	0.09
	Within Groups		5251.10	104	50.49		
	Total		5500.54	106			
CQS Dimension 3 MOT * Gender	Between Groups	(Combined)	50.22	2	25.11	0.83	0.44
	Within Groups		3149.95	104	30.29		
	Total		3200.17	106			
CQS Dimension 4 BEH * Gender	Between Groups	(Combined)	8.80	2	4.40	0.12	0.89
	Within Groups		3836.81	104	36.89		
	Total		3845.61	106			
OE * Gender	Between Groups	(Combined)	50.74	2	25.37	0.21	0.81
	Within Groups		12,283.48	104	118.11		
	Total		12,334.22	106			
TOPI * Gender	Between Groups	(Combined)	2.08	2	1.04	0.17	0.85
	Within Groups		647.94	104	6.23		
	Total		650.02	106			

Note: CIB = Cultural Intelligence—Business (Maximum Performance); CIL = Cultural Intelligence—Leisure (Maximum Performance); CQS = Van Dyne et al. Cultural Intelligence Scale (Typical Performance), Four dimensions of CQS are (1) Metacognitive, (2) Cognitive, (3) Motivational, and (4) Behavioral; OE = Openness to Experience; TOPI = Test of Personal Intelligence.

**Table 3:** Internal-Consistency Reliabilities (Coefficient Alpha).

Test	Coefficient Alpha Reliability	Number of Items
Letter Sets	0.83	15
Figure Classification	0.95	112
Cultural Intelligence—Business (CIB)	0.79	10
Cultural Intelligence—Leisure (CIL)	0.77	9
Cultural Intelligence Combined (CIB+CIL)	0.87	19
Cultural Intelligence Scale (Typical Performance)	0.91	20
Openness to Experience (CQS)	0.79	24
Test of Personal Intelligence Mini (TOPI)	0.78	12

**Table 4:** Intercorrelations.

		1	2	3	4	5	6	7	8	9	10	11	12	13	14	15	16	17	18	19	20
1	Age	1.00	−0.41 **	−0.02	0.08	−0.17	−0.01	−0.17	−0.09	−0.08	−0.09	−0.09	0.12	0.13	0.06	0.08	0.11	0.07	−0.11	0.04	0.08
2	ACT	−0.41 **	1.00	0.68 **	0.78 **	0.95 **	0.34 *	0.51 **	0.11	0.21	0.23	0.24	−0.03	−0.01	0.05	−0.09	−0.06	−0.04	0.26 *	−0.28 *	0.12
3	SAT Reading	−0.02	0.68 **	1.00	0.59 **	0.88 **	0.38 *	0.21	0.13	0.21	0.28 *	0.26 *	−0.09	−0.04	−0.05	−0.10	−0.08	0.06	0.18	−0.16	−0.14
4	SAT Math	0.08	0.78 **	0.59 **	1.00	0.76 **	0.47 **	0.37 **	0.21	0.08	0.15	0.12	0.03	0.03	0.08	−0.11	0.07	−0.17	0.07	−0.35 **	0.20
5	SAT/ACT	−0.17	0.95 **	0.88 **	0.76 **	1.00	0.40 **	0.33 **	0.10	0.05	0.10	0.08	0.03	0.07	0.04	−0.02	−0.01	0.05	0.19	−0.19	0.08
6	GPA	−0.01	0.34 *	0.38 *	0.47 **	0.40 **	1.00	0.17	0.01	0.15	0.18	0.18	−0.06	−0.07	−0.08	0.09	−0.11	−0.25 *	0.03	−0.25 *	0.14
7	LS	−0.17	0.51 **	0.21	0.37 **	0.33 **	0.17	1.00	0.38 **	0.41 **	0.43 **	0.45 **	0.05	0.17	−0.02	−0.01	0.06	0.14	.53 **	−0.08	0.21 *
8	FC	−0.09	0.11	0.13	0.21	0.10	0.01	0.38 **	1.00	0.34 **	0.33 **	0.36 **	−0.04	0.00	0.02	−0.17	0.01	−0.07	0.15	−0.18	0.08
9	CIB	−0.08	0.21	0.21	0.08	0.05	0.15	0.41 **	0.34 **	1.00	.74 **	0.94 **	0.09	0.16	−0.11	0.16	0.14	0.18	0.31 **	0.00	0.15
10	CIL	−0.09	0.23	0.28 *	0.15	0.10	0.18	0.43 **	0.33 **	0.74 **	1.00	0.92 **	0.14	0.15	0.01	0.19 *	0.10	0.11	0.31 **	0.08	0.15
11	CI Total	−0.09	0.24	0.26 *	0.12	0.08	0.18	0.45 **	0.36 **	0.94 **	0.92 **	1.00	0.12	0.17	−0.05	0.19	0.13	0.16	0.33 **	0.04	0.17
12	CQS	0.12	−0.03	−0.09	0.03	0.03	−0.06	0.05	−0.04	0.09	0.14	0.12	1.00	0.74 **	0.73 **	0.80 **	0.76 **	0.26 **	0.03	0.31 **	0.11
13	CQSDimension 1: Metacognitive	0.13	−0.01	−0.04	0.03	0.07	−0.07	0.17	0.00	0.16	0.15	0.17	0.74 **	1.00	0.34 **	0.56 **	0.52 **	0.34 **	0.16	0.26 **	0.17
14	CQSDimension 2: Cognitive	0.06	0.05	−0.05	0.08	0.04	−0.08	−0.02	0.02	−0.11	0.01	−0.05	0.73 **	0.34 **	1.00	0.39 **	0.30 **	0.11	−0.11	0.14	0.07
15	CQSDimension 3: Motivational	0.08	−0.09	−0.10	−0.11	−0.02	0.09	−0.01	−0.17	0.16	0.19 *	0.19	0.80 **	0.56 **	0.39 **	1.00	0.52 **	0.22 *	0.09	0.28 **	0.14
16	CQSDimension 4: Behavioral	0.11	−0.06	−0.08	0.07	−0.01	−0.11	0.06	0.01	0.14	0.10	0.13	0.76 **	0.52 **	0.30 **	0.52 **	1.00	0.19	0.04	0.27 **	−0.03
17	OE	0.07	−0.04	0.06	−0.17	0.05	−0.25 *	0.14	−0.07	0.18	0.11	0.16	0.26 **	0.34 **	0.11	0.22 *	0.19	1.00	0.11	0.01	−0.10
18	TOPI	−0.11	0.26 *	0.18	0.07	0.19	0.03	0.53 **	0.15	0.31 **	0.31 **	0.33 **	0.03	0.16	−0.11	0.09	0.04	0.11	1.00	−0.24 *	0.11
19	CCE	0.04	−0.28 *	−0.16	−.35 **	−0.19	−.25 *	−0.08	−0.18	0.00	0.08	0.04	0.31 **	0.26 **	0.14	0.28 **	0.27 **	0.01	−0.24 *	1.00	−0.01
20	County Visited	0.08	0.12	−0.14	0.20	0.08	0.14	0.21 *	0.08	0.15	0.15	0.17	0.11	0.17	0.07	0.14	−0.03	−0.10	0.11	−0.01	1.00

* *p <* 0.05; ** *p <* 0.01.

**Table 5:** Rotated Principal-Components Matrix: Psychometric assessments, Cultural Intelligence Tests—Business (CIB), Cultural Intelligence Tests—Business (CIL), Cultural Intelligence Scale (CQS), Openness to Experience (OE), and Test of Personal Intelligence (TOPI).

	Component	
	1	2
Total Letter Sets	0.76	0.04
Total Figure Classification	0.61	−0.32
Total CIB Score	0.80	0.17
Total CIL Score	0.80	0.15
Total CQS Score	0.04	0.76
Total OE Score	0.11	0.77
Total TOPI Score	0.61	0.08
Extraction Method: Principal Component Analysis		
Rotation Method: Varimax with Kaiser Normalization		
Rotation converged in 3 iterations		

Notes: Two principal components had Eigenvalues greater than 1. Component 1 had an Eigenvalue of 2.66, accounting for 38.0% of the variance in the data. Component 2 had an Eigenvalue of 1.28, accounting for 18.3% of the variance in the data. The cumulative percent variance accounted for was 56.4%.

**Table 6:** Rotated Principal—Factor Matrix: Psychometric assessments, Cultural Intelligence Tests—Business (CIB), Cultural Intelligence Tests—Business (CIL), Cultural Intelligence Scale (CQS), Openness to Experience (OE), and Test of Personal Intelligence (TOPI).

	Component	
	1	2
Total Letter Sets	0.66	0.06
Total Figure Classification	0.49	−0.18
Total CIB Score	0.76	.21
Total CIL Score	0.76	0.18
Total CQS Score	0.04	0.44
Total OE Score	0.08	0.57
Total TOPI Score	0.48	0.09
Extraction Method: Principal Axis Factoring		
Rotation Method: Varimax with Kaiser Normalization		
^a^ Rotation converged in 3 iterations		

Notes: Two principal factors had Eigenvalues greater than 1. Factor 1 had an Eigenvalue of 2.66, accounting for 38.0% of the variance in the data. Factor 2 had an Eigenvalue of 1.28, accounting for 18.3% of the variance in the data. The cumulative percent variance accounted for was 56.4%.

**Table 7:** Rotated Principal—Component Matrix: Cultural Intelligence Tests—Business (CIB), Cultural Intelligence Tests—Business (CIL), Cultural Intelligence Scale (CQS), Openness to Experience (OE), Test of Personal Intelligence (TOPI), and SAT to ACT conversion score.

	Component		
	1	2	3
Total CIB Score	0.90	0.11	0.05
Total CIL Score	0.89	0.08	0.08
Total CQS Score	0.09	0.76	−0.07
Total OE Score	0.05	0.76	0.08
Total TOPI Score	0.38	−0.20	0.61
SAT/ACT	−0.08	0.14	0.88
Extraction Method: Principal Component Analysis			
Rotation Method: Varimax with Kaiser Normalization			
Rotation converged in 5 iterations			

Notes: Three principal components had Eigenvalues greater than 1. Component 1 had an Eigenvalue of 1.93, accounting for 32.1% of the variance in the data. Component 2 had an Eigenvalue of 1.20, accounting for 20.0% of the variance in the data. Component 3 had an Eigenvalue of 1.05, accounting for 17.4% of variance in the data. The cumulative percent variance accounted for was 69.5%.

**Table 8:** Rotated Principal—Factor Matrix: Cultural Intelligence Tests—Business (CIB), Cultural Intelligence Tests—Business (CIL), Cultural Intelligence Scale (CQS), Openness to Experience (OE), Test of Personal Intelligence (TOPI), and SAT to ACT conversion score.

	Factor		
	1	2	3
Total CIB Score	0.86	0.14	0.09
Total CIL Score	0.77	0.12	0.15
Total CQS Score	0.06	0.46	−0.02
Total OE Score	0.06	0.44	0.04
Total TOPI Score	0.26	−0.10	0.43
SAT/ACT	0.01	0.07	0.47
Extraction Method: Principal Axis Factoring			
Rotation Method: Varimax with Kaiser Normalization			
Rotation converged in 5 iterations			

Notes: Three principal factors had Eigenvalues greater than 1. Factor 1 had an Eigenvalue of 1.93, accounting for 32.1% of the variance in the data. Factor 2 had an Eigenvalue of 1.20, accounting for 20.0% of the variance in the data. Factor 3 had an Eigenvalue of 1.05, accounting for 17.4% of variance in the data. The cumulative percent variance accounted for was 69.5%.

## Data Availability

The data are available from Chak Haang Wong, who helped to collect the data and who analyzed the data.

## Appendix A. Sternberg Cultural Intelligence Test (SCIT)

Instructions: Please read the following information and come up with a solution to solve the problems in scenarios and alternative solutions if the main one does not work out.

### Appendix A.1. Part I

You have just arrived on your current confidential assignment in a foreign country with which you are largely unfamiliar. Your assignment is to negotiate a memorandum of agreement between your organization and a large organization in the foreign country. You were told that you were expected to return to the US with a signed agreement. Before leaving the US, you were given very little information about your destination country, and most of that was basic information on the political system, imports and exports, and the general economy. You do not know the language and you know that relations with the country are tense. You realize that your room in the hotel in which you are staying has no access to the World Wide Web. Moreover, your cell phone does not work in this country.

1. You have finally arrived, and you are ready to conduct your business. You go to the office to which you were told to report. You announce yourself to an assistant. He leads you through a door to an individual who looks like a high-level administrator. She says “Hello” to you in perfect English. You explain why you are there. To your astonishment, she laughs in your face. *What would you do?*

2. You have important sensitive and confidential information to convey back to your superiors. When you go down to the receptionist, you are told that there is no problem. You can use a hotel computer, which is connected to the Worldwide Web. Or, she says, you can use the phone in your room and she can connect you to wherever you need to be connected. She then leads you to a computer in the business center. *What would you do?*

3. Some of the people at the dinner speak English; others don’t. After the meal, there is a lot of drinking as people socialize. You try your best to control your drinking. But you let a joke slip out about the leader of the country. You realize you should not have made the joke and that you would not have if you had not had a few too many drinks. You can tell by people’s faces that you have committed a faux pas, maybe a serious one. *What would you do?*

4. One of the organizational executives with whom you are supposed to negotiate a deal hands you another a glass of the national drink, which is very strong. He smiles and obviously expects you to drink it. Other people from the organization are standing by, watching, and nodding approvingly. You know you already have drunk way too much. *What would you do?*

5. Two nights later, at an organizational party, you are introduced to a very attractive individual. It is pretty obvious to you that the introduction was not incidental but rather was planned. The individual shows great interest in you, perhaps, you think, too great, given that you are strangers. The introducer smiles and puts a finger over his lips. He seems to you to be saying that “What happens at the party stays at the party,” but you really can’t be sure. *What would you do?*

6. At the end of the party, an executive from the company with which you are supposed to negotiate the memorandum of agreement quickly and unexpectedly slips you a very substantial amount of money, smiles, and tells you to enjoy yourself. The money is now in your hand. *What would you do?*

7. You are negotiating with a high-level official in the organization with which you need to sign an agreement. No matter what you say, she seems to disagree. You feel that the negotiation is not going well and that it really doesn’t matter what you say, she will disagree. You really want and need for this negotiation to succeed. *What would you do?*

8. During your last day on the assignment, the organization with which you are negotiating asks you to sign a form. They explain to you that the form is simply an acknowledgment that you negotiated with them. But the document is in the language of the country, which you do not know, and no one seems willing to explain it in any detail. You really need this negotiation to work out. *What would you do?*

9. On your last night on assignment, there is a knocking on your hotel door. You open the door and two men in what appear to be military or police uniforms are standing facing you. They explain to you in perfect English that they need to search your room, as is standard procedure before someone leaves the country. No one ever told you about any standard room searches. You have highly confidential documents in the room but no contraband or anything that, to your knowledge, would cause legal problems. *What would you do?*

10. As you get ready to approach customs at the airport, a woman seems to come out of nowhere and approaches you. You think you recognize her from your trip. You can’t quite place her but believe she was one of the employees of the organization with which you negotiated. She says that the organization forgot to give you a farewell gift and that she was instructed to give it to you before you departed. She has only now caught up with you. She shoves a gift box into your hands. It is packed in gift wrap with a gold ribbon but otherwise has no identifying marks. On the one hand, you don’t want to insult the organization but, on the other hand you have no idea what is in the box. *What would you do?*

### Appendix A.2. Part II

Instructions: Please read the following information and come up with a solution to solve the problems in the scenarios and alternative solutions if the main one does not work out.

You have earned a three-week well-deserved holiday, and you have decided to travel abroad and go on an adventure in an unfamiliar country. The country is located in a very remote and isolated location. You know very little about the country. You do not understand or speak the language, and you have never experienced the culture there or encountered people from that country. Moreover, there is no worldwide internet access, and your cell phone does not work in this country. But as a person interested in exploring and challenging yourself, you are excited about the upcoming trip. The departure date is soon approaching!

1. You have just arrived after three grueling flight connections. You are exhausted and desperately searching for a taxi to get to the hotel. As you are looking around in the airport, a man approaches you and gestures to lead you. You do not know who he is or understand what he is saying. *What would you do?*

2. You finally get a ride to the hotel, and when you get to the destination and ask how much you need to pay, the driver stretches out his second and third fingers. To your understanding, this means “two,” so you give him 20 dollars in the local currency. However, the driver looks angry and starts to shout, apparently asking for more money. You feel like he is trying to rip you off, as this 10-min trip certainty could not cost more than 20 bucks. *What would you do?*

3. As you enter the hotel to settle down, you notice that the hotel staff are raising their hands up in the air with their palms facing toward you. You do not know what this gesture means. *What would you do?*

4. You are excited to try the local food, so you randomly pick a restaurant near the hotel. You cannot read and understand the menu. You want to order food, but you also want to convey your likes and dislikes to the waiter, who seems not to speak any English. Hoping for the best, you ask for “fish.” He smiles and writes down nothing. *What would you do?*

5. After dinner, you begin to have a stomachache. You are worried that, somehow, you might have eaten something to which you are allergic or even have gotten food poisoning. You think you better go to a hospital, but none of the signs on the street are in English nor has a picture that looks like a hospital. *What would you do?*

6. After coming back from the hospital, you take a nice shower and are ready for a good night’s rest. On the next day, you plan to take a walk in the city and take some pictures of the unique architectures. You walk into a small alley that is nowhere like where you are from, as you are curious to see how the local people are living next to the alleys. After a while, you realize both that you are lost, and that people are giving you strange looks. They are saying words you do not understand, yet you feel a sense of hostility and even threat in their tone and facial expression. A group of young, rough-looking men approaches, looking menacing. *What would you do?*

7. A few days later, you sign up for a tour to go on a hike in the woods. After an uncomfortable six hours of riding a bus, you make friends with a couple, who are also visitors from the same country as you. You are happy to talk to someone in your native language, and the couple even teach you a few words in the local language. You and the tour group hike for three hours and then stop for quick snack. The tour guide hands you what might be the most disgusting food you ever have seen. It looks like it comprises wriggly worms that might even be alive. Whatever they are, they smell no better than they look. You hesitate to eat it, but the rest of the group seems to be enjoying the snack. You notice other people staring at you. *What would you do?*

8. It is the last day of your trip. You cannot wait to go home but, at the same time, you feel a little bit sad about leaving. You go to a bar and order the local beer that you like. You start to feel tipsy and happy while talking to a couple of other foreigner visitors. However, when it is time to pay the check, you realize that your wallet is gone. Luckily, you put your passport in a separate bag, so it is not stolen; however, all your cash, bank cards, and driver’s license are gone. *What would you do?*

9. The next day you arrive at the airport for your flight home. You are waiting to go through a customs inspection, which you believe should be non-problematical because you bought nothing during the trip that you are taking home. You then see a sign, which comes as a total surprise, that says that you are allowed to check in only one piece of luggage. You have two big suitcases with many souvenirs for friends and family, and they are probably overweight. You really cannot easily afford to throw the luggage away. *What would you do?*
